# Recent Applications of Chitosan and Its Derivatives in Antibacterial, Anticancer, Wound Healing, and Tissue Engineering Fields

**DOI:** 10.3390/polym16101351

**Published:** 2024-05-10

**Authors:** Saeid Mezail Mawazi, Mohit Kumar, Noraini Ahmad, Yi Ge, Syed Mahmood

**Affiliations:** 1 School of Pharmacy, Management and Science University, Shah Alam 40100, Selangor, Malaysia; saeidmezail@yahoo.com; 2 Department of Pharmaceutical Sciences and Technology, Maharaja Ranjit Singh Punjab Technical University (MRSPTU), Bathinda 151001, Punjab, India; maddysharma3303@gmail.com; 3 Department of Chemistry, Faculty of Science, Universiti Malaya, Kuala Lumpur 50603, Malaysia; ainie@um.edu.my; 4 School of Pharmacy, Queen’s University Belfast, Belfast BT9 7BL, UK; 5 Department of Pharmaceutical Technology, Faculty of Pharmacy, Universiti Malaya, Kuala Lumpur 50603, Malaysia

**Keywords:** chitosan, pharmaceutical, applications, cancer, tissue engineering, drug delivery, MeSH

## Abstract

Chitosan, a versatile biopolymer derived from chitin, has garnered significant attention in various biomedical applications due to its unique properties, such as biocompatibility, biodegradability, and mucoadhesiveness. This review provides an overview of the diverse applications of chitosan and its derivatives in the antibacterial, anticancer, wound healing, and tissue engineering fields. In antibacterial applications, chitosan exhibits potent antimicrobial properties by disrupting microbial membranes and DNA, making it a promising natural preservative and agent against bacterial infections. Its role in cancer therapy involves the development of chitosan-based nanocarriers for targeted drug delivery, enhancing therapeutic efficacy while minimising side effects. Chitosan also plays a crucial role in wound healing by promoting cell proliferation, angiogenesis, and regulating inflammatory responses. Additionally, chitosan serves as a multifunctional scaffold in tissue engineering, facilitating the regeneration of diverse tissues such as cartilage, bone, and neural tissue by promoting cell adhesion and proliferation. The extensive range of applications for chitosan in pharmaceutical and biomedical sciences is not only highlighted by the comprehensive scope of this review, but it also establishes it as a fundamental component for forthcoming research in biomedicine.

## 1. Introduction

Biopolymer chitosan is derived from the natural polymer chitin found in the exoskeletons of crustaceans like shrimp and crabs [1,2]. Over the past few years, chitosan has garnered significant recognition for its numerous potential applications in cutting-edge pharmaceutical research. With its biocompatibility, biodegradability, and capacity to improve drug delivery, chitosan has been the subject of substantial research as a drug carrier in the pharmaceutical field [3]. The discovery and development of chitosan have been fascinating to see unfold over time, and scientists continue to find new and interesting ways to put this multipurpose substance to use. It has been known for years that chitin is a versatile material; nevertheless, in the middle of the twentieth century, researchers discovered chitosan and started investigating its possible applications. Chitin has been acknowledged for a considerable time as a highly adaptable material [4]. Researchers investigating the bioactivity of chitosan used various dosage forms, including chitosan nanoparticles [5], chitosan hydrogels [6], chitosan films [7], and chitosan microspheres [8]. The anti-inflammatory and antimicrobial characteristics of chitosan and its capacity to stimulate cell growth and blood vessel formation give it a leg up in the realm of wound healing. Chitosan has been employed as a scaffold material in tissue engineering to promote cell adherence and proliferation [9]. The antibacterial effects of chitosan result from the molecule’s capacity to cause membrane disruption in microorganisms, such as bacteria and fungi. Currently, chitosan is used in several sectors, such as the pharmaceutical, food and beverage, cosmetics, and agricultural sectors [3]. It is used as a natural preservative in food goods, a wound dressing due to its antimicrobial characteristics, and as a nutritional supplement for weight loss and cholesterol reduction. This article presents an outline of chitosan used in contemporary pharmaceutical research, demonstrating it as a biomaterial with therapeutic promise. Drug delivery, wound healing, tissue engineering, and antibacterial activity are some of the areas this review hopes to shed light on. Furthermore, this comprehensive analysis updates the application of chitosan in the modern pharmaceutical industry, including the current developments. Recent progress in how the pharmaceutical industry employs chitosan has unveiled its potential in various domains. Notably, the emergence of the antibacterial activity of chitosan underscores its potential role in combating microbial infections, while its incorporation in tissue engineering showcases its ability to improve cellular adhesion and proliferation. Furthermore, the utilisation of chitosan in wound healing and cancer investigations highlights its multifunctional attributes, including its ability to expedite wound recovery and its potential as a drug carrier and adjuvant in cancer treatment. In light of the increasing worldwide emphasis on sustainability and environmental footprint reduction, there has been a substantial surge in the need for biodegradable and naturally derived materials in numerous industries, including pharmaceuticals and biomaterials. Chitosan is an exceptional material in this context on account of its biocompatibility, biodegradability, and absence of toxicity. These characteristics not only promote the utilisation of chitosan in biomaterial applications and pharmaceutical formulations but also correspond to the overarching goal of creating environmentally friendly solutions in the fields of science and technology. The incorporation of chitosan and its derivatives into pharmaceutical products holds the promise of catalysing a more environmentally friendly and sustainable future. This underscores the urgent need for transitioning towards materials that offer superior therapeutic efficacy while minimising environmental impacts. As industries increasingly prioritise sustainability, chitosan emerges as a pivotal player, symbolising a shift towards greener and more responsible practices in the quest for innovative solutions.

### 1.1. Why Chitosan–Highly Versatile and Widely Acceptable

**Figure 1:**
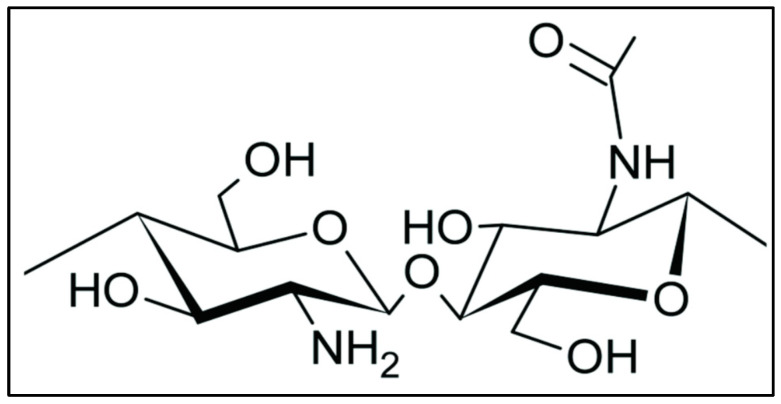
Chemical structure of chitosan: this figure shows the ability of chitosan to have a different functional group that gives diverse applications of chitosan.

Chitosan has received substantial interest in a variety of scientific domains owing to its distinct qualities and favourable traits that make it superior to other polymers. Chitosan stands out from other biopolymers due to its outstanding qualities such as biocompatibility, biodegradability, and adaptability. Chitosan has a major edge over other polymers in terms of biocompatibility [10]. Chitosan is highly compatible with biological systems, including cells and tissues, making it the optimal material for biomedical applications. Chitosan’s biocompatibility allows it to be employed in a variety of applications, including drug delivery systems, tissue engineering scaffolds, wound healing dressings, and implant coatings, without generating adverse responses or causing major damage to the host organism. Chitosan’s biodegradability is another significant feature [11]. This feature exists because it is made from chitin, a naturally occurring polymer found in crab exoskeletons and fungal cell walls. Chitosan undergoes enzymatic decomposition by lysozymes and other microbial enzymes when exposed to suitable environmental conditions, resulting in non-toxic degradation products. This property is especially useful in situations where polymer persistence is undesired, such as ecologically friendly packaging materials or controlled release systems. In addition, the adaptability of chitosan adds to its excellence as a polymer [12]. To improve its qualities and adjust its performance for particular applications, it may be readily manipulated by chemical derivatisation or physical alterations. Chitosan’s amino groups, for example, may be chemically changed to incorporate functional groups or to change their solubility and charge properties. Such alterations broaden chitosan’s potential uses, enabling it to be used in a variety of disciplines such as food science, medicine, agriculture, and wastewater treatment (Figure 1, the chemical structure of chitosan). In summary, chitosan outperforms other polymers in terms of biocompatibility, biodegradability, and adaptability. Because of these qualities, chitosan is an appealing material for a broad variety of applications, notably in the biomedical area. Furthermore, because of its eco-friendliness and widespread availability, chitosan is a good option for sustainable solutions in a variety of sectors [13,14,15,16].

### 1.2. History of Chitosan

The shells of crabs, lobsters, prawns, and crayfish were decalcified by Hatchett in 1799 using mineral acids; he noted that the process resulted in a moderate effervescence. Later, the observation was made that the decalcified shells were soft and plastic, yellowish in colour, and like cartilage, retaining the original figure. Scientists studied chitosan for the first time in earnest in the 1930s and 1940s. Japanese researchers then found that deacetylation might transform chitin into the more helpful chitosan. Researchers in Japan and others started investigating chitosan’s applications in the 1960s and 1970s. They discovered that chitosan has several beneficial qualities that make it applicable in many fields, such as water purification, agriculture, and medicine. Water-absorbing, molecule-binding, and gel-forming are just a few of chitosan’s unusual abilities [17].

## 2. Production of Chitosan

**Figure 2:**
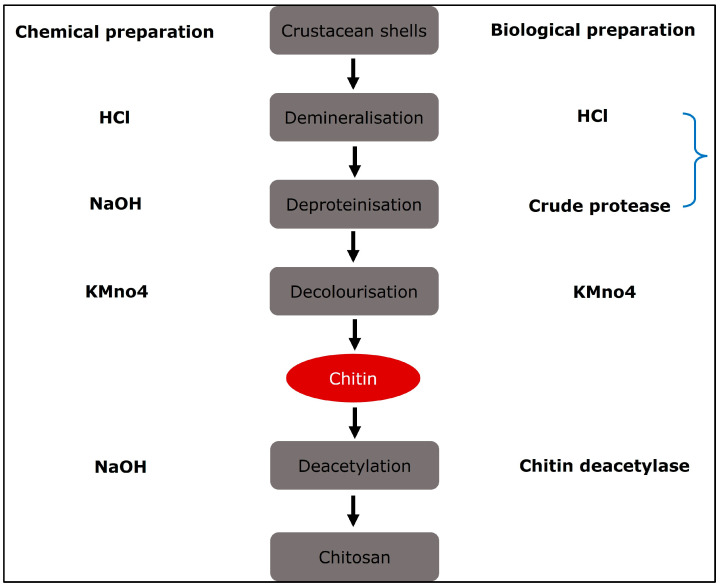
Diagrammatic representation of chitosan preparation from natural source in which natural and chemical processing are utilised.

Chitin is the basic ingredient used in the production of chitosan. The primary sources are crustacean shells, especially crabs and shrimp. Thinner shrimp shells make the purifying process easier. Shells of the same size and species are usually gathered, cleaned, dried, and crushed into little shell pieces. There is no standard purification process since various chitin sources require different treatments owing to structural variety [18]. The process is traditionally separated into three steps: demineralisation, deproteinisation, and decolourisation, which can be accomplished by chemical [19] or biological (enzymatic therapy or fermentation) therapies [20]. If the end-products are to be employed for biomedical or pharmaceutical applications, they must be extensively purified, since remaining proteins, minerals, or pigments might cause major adverse effects. Chitin can be converted to chitosan by enzymatic [21] or chemical deacetylation [22]. Because of economic concerns and the capability of mass manufacturing, chemical deacetylation is more typically utilised for commercial preparation. Figure 2 depicts the procedures involved in the chemical and biological synthesis of chitosan from crustacean shells.

### Chitosan Derivatives

Chitosan is indeed a versatile and amenable molecule for chemical modification due to its unique structural properties. Chitosan is composed of repeating units of N-acetylglucosamine (GlcNAc) and glucosamine (GlcN) linked by β-1,4 glycosidic bonds. This polymer contains several functional groups that can be chemically modified without significantly disturbing its degree of polymerisation (DP) [20]. These functional groups include primary amine (-NH2) groups in the glucosamine units. These primary amine groups are highly reactive and can undergo various chemical reactions. They can be acylated, alkylated, or reacted with other reagents to introduce various functional groups, such as carboxyl groups, sulfonate groups, or other chemical moieties [21]. Chitosan also has primary (-OH) and secondary (-OH) hydroxyl groups in its monomers. These hydroxyl groups are also chemically reactive and can be modified through reactions such as esterification, etherification, or oxidation. This allows for the introduction of different functional groups or the modification of the polymer’s physical and chemical properties [21]. Chitosan is extracted from chitin by removing the acetyl group, which requires a harsh process involving concentrated aqueous or alcoholic NaOH solutions, nitrogen purging, or the addition of sodium borohydride to prevent undesirable reactions like depolymerisation and the production of reactive species. Efforts have been made to reduce the quantity of NaOH needed for the reaction [23]. A common chemical modification of chitosan includes acetylating chitosan, which involves introducing acetyl groups (-COCH3) onto its amino groups. This modification alters the properties of chitosan, including its solubility, pH sensitivity, and biodegradability. The process typically involves a reaction with acetic anhydride or acetyl chloride under mild conditions [24]. The alkylation of chitosan involves introducing alkyl groups onto its amino groups, typically through reactions with alkyl halides. This process can alter the properties of chitosan, such as its solubility, chemical reactivity, and interaction with other molecules [25,26]. Crosslinked chitosan derivatives are created by crosslinking the primary amine groups of chitosan with crosslinking agents, such as glutaraldehyde or genipin. The crosslinking reaction forms bridges between the primary amine groups of adjacent chitosan molecules, resulting in a three-dimensional network structure with improved mechanical properties and controlled release behaviours [27]. Graft copolymers of chitosan involve attaching other polymer chains onto the primary amine groups of chitosan through grafting reactions, such as free radical polymerisation or enzymatic grafting. The primary amine groups of chitosan act as anchor points for grafting polymer chains, leading to the formation of copolymers with altered properties, such as enhanced mechanical strength or controlled release capabilities [28]. By adjusting the degree of deacetylation (removing acetyl groups), the solubility of chitosan can be controlled [29].

These chemical modifications enable chitosan to be tailored for various applications, including drug delivery, wound healing, water treatment, and more. The adjustable creativity of chitosan in chemical modification makes it a valuable material in the fields of biotechnology, pharmaceuticals, and materials science [30]. Some of the common examples of modified chitosan are shown in Table 1.

## 3. Advantages and Limitations

Chitosan and its derivatives hold immense promise for wound healing and tissue engineering applications, offering a plethora of advantages coupled with certain limitations that warrant consideration. One of the primary advantages lies in their biocompatibility and biodegradability, ensuring minimal adverse reactions and gradual degradation within the body, respectively [53]. This property is crucial for scaffolds and implants used in tissue engineering, as it facilitates integration with host tissues without eliciting harmful immune responses [54]. Moreover, chitosan and its derivatives possess inherent bioactivity, promoting cell adhesion, proliferation, and differentiation, which are essential processes for tissue regeneration [55]. Their antimicrobial properties further contribute to wound healing by preventing infections, thereby facilitating the healing process. Additionally, the mechanical properties of chitosan-based materials can be tailored to mimic those of native tissues, providing structural support where needed [56]. However, it is important to acknowledge certain limitations, such as the relatively low mechanical strength of chitosan scaffolds, which may restrict their use in load-bearing applications. Furthermore, while chitosan exhibits controlled drug delivery capabilities, its release kinetics may not always match the desired therapeutic requirements, necessitating further optimisation [57,58]. Additionally, the immunogenicity of chitosan and its derivatives in some individuals may pose challenges in certain clinical settings [59]. Despite these limitations, the numerous advantages of chitosan and its derivatives make them valuable tools in wound healing and tissue engineering, with ongoing research aimed at addressing and overcoming their limitations to unlock their full potential in regenerative medicine [60,61].

## 4. Bioactivities of Chitosan

Chitosan and its derivatives have a wide range of biological implementation. Chitosan performs well against a range of yeasts, filamentous fungus, and bacteria [53]. An overview of the bioactivities of chitosan is given in the section below.

### 4.1. Anti-Inflammatory and Antimicrobial Activity of Chitosan

The investigation of chitosan’s anti-inflammatory and antimicrobial properties has attracted considerable interest in recent years. Chitosan has the potential to combat inflammation and microbial infections, making it a promising therapeutic agent. According to a previously published paper, chitosan may have either pro- or anti-inflammatory effects, depending on the concentration, administration, and dosage form utilised [62,63]. An inflammatory response may be triggered by chitosan because it stimulates the innate immune system, which includes macrophages, neutrophils, and dendritic cells [64]. Pro-inflammatory cytokines such as tumour necrosis factor-alpha (TNF-), interleukin-1 beta (IL-1), and interleukin-6 (IL-6) are produced as a result of this activation [65,66]. Its pro-inflammatory action is beneficial for wound healing because it stimulates the growth of new blood vessels and the migration of immune cells to the site of damage. On the other hand, chitosan has been shown to have anti-inflammatory properties by reducing the production of inflammatory cytokines and chemokines such tumour necrosis factor-alpha, interleukin-1 beta, interleukin-6, and monocyte chemoattractant protein-1 (MCP-1) [67]. The capacity of chitosan to interact with Toll-like receptors (TLRs) on immune cells, which play a role in the detection of microbial components and the onset of the inflammatory response, has been credited with this anti-inflammatory effect [68]. The transcription nuclear factor kappa B (NF-B), which controls the production of pro-inflammatory genes, is an example of a signalling pathway that chitosan has been demonstrated to influence [69]. The antioxidant activity may enhance the anti-inflammatory properties chitosan has been shown to possess. Its antioxidant effect is due to chitosan’s capacity to scavenge reactive oxygen species (ROS), which have a role in both the development and perpetuation of inflammation [70]. Reports of both pro-inflammatory and anti-inflammatory actions of chitosan indicate that its inflammatory activity is nuanced and situational [71]. The inflammatory activity caused by chitosan may be affected by concentration, formulation, immune cell type, and microenvironment factors.

Antimicrobial activity investigations have shown that chitosan is effective against various microorganisms, including bacteria, yeasts, moulds, and viruses [72,73]. *Escherichia coli*, *Staphylococcus aureus*, *Salmonella*, *Candida albicans*, and *Aspergillus niger* are some of the pathogenic microorganisms that chitosan has been demonstrated to be effective against [74]. Modifying chitosan’s physical and chemical characteristics, such as raising its degree of deacetylation, molecular weight, and concentration, may boost its antibacterial effectiveness [75]. The antibacterial efficacy of chitosan has been shown for a number of different chitosan preparations, such as nanoparticles, films, hydrogels, and chitosan-coated surfaces [73,76,77]. There has been research on using chitosan as a natural preservative in food and agricultural products to slow the development of microorganisms and lengthen their storage life [78]. In Table 2, some examples of chitosan-based formulations for antibacterial activity are discussed.

### 4.2. Latest Developments in Chitosan’s Antimicrobial Activity

Recent research highlights chitosan’s antimicrobial potential, particularly in creating new agents as an alternative to traditional antibiotics amid rising antimicrobial resistance. Elmehbad et al. (2022) introduced a novel approach to chemically modified chitosan using salicylhydrazide, yielding two derivatives: salicylhydrazide chitosan Schiff’s base (SCsSB) and salicylhydrazide chitosan (SCs). Further modifications produced SCs/TiO_2_ nanocomposites with enhanced antibacterial and anti-biofilm efficacy, particularly SCs/TiO_2_-3%, which outperformed standard antibacterials and showed specific activity based on microbial cell wall structures. This work suggests modified chitosan derivatives as potent biomedical tools with applications across various biological domains, indicating their safety for human cells [79].

A pivotal study in biopolymer biomedical applications successfully synthesised a bioactive chitosan Schiff base (CTS-SB) derivative, enhancing its antibacterial and antidiabetic properties. This derivative outperformed pure chitosan in ion exchange capacity and showed significant antibacterial activity against both Gram-positive and Gram-negative bacteria, including *E. coli* and *S. aureus*. The study also revealed CTS-SB’s potential anticancer activity and compatibility with human skin fibroblasts, underscoring its suitability for advanced biomedical applications, such as drug delivery and wound healing [80].

**Table 2:** Evaluation of antimicrobial activity of chitosan-based formulations against various microorganisms, with special observations and supporting references.

S. No.	Type of Formulation	Size of NP/Fibres	Microbes	Special Comments	References
1.	*Bletilla striata* (BSP) film	0.097 ± 0.004 to 0.136 ± 0.003 mm thickness	*Escherichia coli* *staphylococcus aureus* and *Pseudomonas* *aeruginosa*	In this study, the author reported that the prepared BSP/CS films may be used to create biomaterials for new wound dressings.	[81]
2.	Eucalyptus-oil-loaded chitosan nanofibres	48.26 nm	*Staphylococcus aureus*	The developed nano-chitosan/Eucalyptus oil/cellulose acetate nanofibre has excellent antimicrobial properties and shows promise as a wound healing dressing.	[82]
3.	CSNPs	141.20 nm	*S. aureus* and *P. aeruginosa*	The dose-dependent antibacterial activity was observed.	[83]
4.	Lecithin-coated CSNPs	235 ± 20 nm	*S. aureus*	A 2-fold decrease in the survival rate of *S. aureus* was observed.	[84]
5.	CS-AgNPs	10–30 nm	*Methicillin-resistant streptococcus aureus (MRSA)*	MRSA was not detected in all treated groups.	[85]
6.	CSNPs	408.30 ± 53.17 nm	*S. aureus*	More than 90% inhibition rate was observed in CSNP-treated groups.	[86]
7.	AgNPs	-	*S. aureus*, *P. aeruginosa*, and *E. coli*	The prepared sponges loaded with AgNPs show excellent antibacterial activity against *S. aureus*, *P. aeruginosa*, and *E. coli*.	[87]
8.	CS-AgNPs	10–50 nm	*MRSA* and *P. aeruginosa*	The CS-AgNPs show good antibacterial activity against MRSA and *P. aeruginosa*.	[88]
9.	CS-AgNPs	22.80 nm	*E. coli*	The CS film loaded with CS-AgNPs showed higher inhibition against *E. coli* compared to CS-AgNP solution.	[89]
10.	CS fibres	-	*Escherichia coli*, *Staphylococcus aureus*, and *Candida albicans*	The prepared fibres showed excellent antibacterial activity.	[90]
11.	CS nanofibres	140–170	*E. coli* and *S. aureus*	The CS nanofibres loaded with cinnamon extract enhance the antibacterial activity.	[91]
12.	CSNPs	208.40 ± 15.70 nm	*E. coli* and *S. aureus*	The CSNPs show dose-dependent antibacterial activity and have greater effect against *S. aureus* than *E. coli*.	[92]
13.	CSNPs	51.67 ± 12.55 nm	*S. aureus*, *P. aeruginosa*, *E. coli*, *B. subtilis*, and *C. albicans*	The CSNP loaded with SSD show greater effect against gram +ve bacteria than gram −ve bacteria.	[93]
14.	AgNPs	5–10 nm	*Staphylococcus aureus* and *Pseudomonas aeruginosa*	The 3D scaffold loaded with AgNPs showed excellent antibacterial activity.	[94]

*E. coli*—*Escherichia coli*; *S. aureus*—*Staphylococcus aureus*; *C. albicans*—*Candida albicans*; SSD—silver sulfadiazine; CS—chitosan; AgNPs—silver nanoparticles; MRSA—*Methicillin-resistant Staphylococcus aureus*.

#### Antibacterial Mechanism of Action of Chitosan

**Figure 3:**
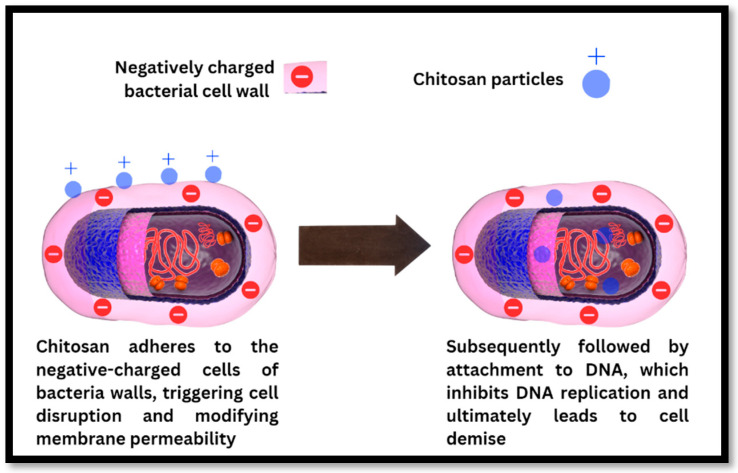
Mechanism of antibacterial action of chitosan particles on the bacterial cell wall leading to the disruption of the bacterial cell wall, binding to the bacterial DNA and stopping DNA replication, resulting in bacterial cell death.

The precise mechanism is not completely understood, but it is believed that multiple independent factors are involved. Chitosan adheres to the negative-charged cells of bacterial walls, triggering cell disruption and modifying membrane permeability, according to one proposed mechanism. This is subsequently followed by attachment to DNA, which inhibits DNA replication and ultimately leads to cell demise (Figure 3). Alternatively, chitosan may inhibit microbial proliferation by acting as a chelating agent that selectively binds to trace metal elements. The polycationic structure of chitosan influences its antibacterial activity. At acidic pH levels, the electrostatic interaction between positively charged chitosan and negatively charged bacterial surface components is essential to its antibacterial activity [27,37]. Antibacterial activity is proportional to the positive charge density of chitosan, with greater positive charge densities leading to greater antibacterial activity. The number of amino groups on the chitosan backbone also affects the electrostatic interaction, since a greater number of amino groups increases antibacterial activity. In addition, the size and shape of chitosan particles can influence their mode of action, with larger particles engaging in the cell surface and changing cell permeability [38,64,66]. Chitosan-based networks, including chitosan microspheres, can manifest antibacterial activity by disrupting the surface of the bacterial cell or building an impermeable layer surrounding the bacterial cell, thereby preventing critical movement and leading to cell death. Chitosan and its derivatives possess antimicrobial properties and have been studied extensively for a variety of applications in healthcare, food, agriculture, and other industries. To thoroughly comprehend the mechanisms underlying chitosan’s antibacterial activity and to investigate its potential applications, additional research is required [73].

### 4.3. Wound Healing Activity of Chitosan

Chitosan’s unique features, such as biocompatibility, biodegradability, and non-toxicity, are responsible for its wound healing action. The healing process may be sped up with the help of chitosan since it stimulates cell growth, blood vessel formation, and extracellular matrix (ECM) creation [95]. The proliferation of fibroblasts, keratinocytes, and endothelial cells, all of which play a role in wound healing, may be boosted by chitosan. It promotes the repair of injured tissue and quickens the recovery time [96]. New blood vessel growth is critical for delivering oxygen and nutrients to the injured area, and chitosan has been shown to stimulate this process. Growth factors, including vascular endothelial growth factor (VEGF) and basic fibroblast growth factor (bFGF), are responsible for this effect [97]. Chitosan has been shown to increase the production of collagen and other extracellular matrix (ECM) substances (GAGs) [98]. To the mending tissue, these elements provide structural stability and tensile strength. In several experimental and clinical settings, chitosan has been found to hasten and improve wound recovery. Films, hydrogels, and dressings are just some of the shapes it may take to provide a steady stream of chitosan and speed up the healing process [99]. Chitosan has been found to aid in wound healing in several pharmaceutical dosage forms. Chitosan films may be placed directly on the injury to treat wounds. They provide a barrier against infection and help wounds heal by keeping the area wet, encouraging cell proliferation and migration [100,101]. Chitosan hydrogels, which are three-dimensional networks of crosslinked chitosan molecules, readily absorb water.

**Figure 4:**
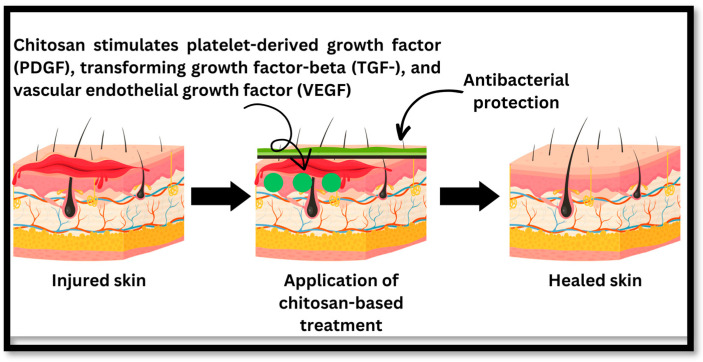
The healing process of injured skin: chitosan inhibits bacterial growth and speeds up the healing process through healing factors such as platelet-derived growth factor (PDGF), transforming growth factor-beta (TGF-), and vascular endothelial growth factor (VEGF).

Wound healing is aided by the moist environment provided by these topical treatments [102]. Porous materials like chitosan nanocomposite sponges may be used to pack a wound. They are effective in soaking up wound exudate and keeping the area around the wound wet, which is necessary for healing [103]. Injuries may be treated with the help of chitosan nanoparticles that carry healing agents to the affected area. One option is to apply them directly to the wound [104], while another is to inject them [105]. The direct application of a chitosan spray to a wound is one option. They may be applied as a thin coating on wounds to provide a moist environment that aids healing (Figure 4) [106]. Some examples of chitosan-based formulations for wound healing are shown in Table 3.

#### Mechanism of Wound Healing of Chitosan

The wound healing process is divided into four stages: haemostasis, inflammation, proliferation, and skin remodelling [107,108,109]. The coagulation system is initiated during the haemostasis stage when blood vessels constrict and platelets aggregate. Fibrinogen is converted into insoluble fibrin, which forms clots to control bleeding. During the inflammation stage, inflammatory cells eliminate microorganisms and necrotic tissue. In the proliferation stage, epithelial cells multiply and move to form epithelial tissue to cover the wound. Granulation tissue covers the tissue gap, but no epithelialisation occurs. Fresh epidermis and dermis will regenerate during the last remodelling step to complete the skin restoration operation [110].

Chitosan and its derivatives will mostly contribute to wound healing during the first three phases. Chitosan plays a crucial function in wound healing via a variety of mechanisms. Firstly, chitosan possesses inherent antimicrobial properties that aid in the prevention and treatment of chronic wound infections. Chitosan effectively reduces the likelihood of microbial colonisation and subsequent inflammation by creating a physical barrier against bacteria and fungi. In addition, chitosan’s ability to speed up the production of biological mediators substantially contributes to the wound healing process. The secretion of growth factors such as platelet-derived growth factor (PDGF), transforming growth factor-beta (TGF-), and vascular endothelial growth factor (VEGF) is stimulated by chitosan. These growth factors play crucial roles in the proliferation and migration of cells involved in wound healing, including fibroblasts, endothelial cells, and keratinocytes (Figure 3). Chitosan facilitates the formation of new blood vessels and the repair of damaged tissues by promoting cellular proliferation and angiogenesis. Moreover, chitosan has immunomodulatory properties, which influence the immune response at the lesion site. It stimulates the production of cytokines and chemokines that modulate inflammation and immune cell recruitment, such as interleukin-6 (IL-6) and tumour necrosis factor-alpha (TNF-). The immunomodulatory properties of chitosan contribute to a balanced and regulated inflammatory response, thereby facilitating the resolution of inflammation and fostering tissue repair. Furthermore, the hydrophilic nature of chitosan enables it to absorb surplus wound exudate and maintain a moist environment conducive to healing. This moisture retention facilitates cell migration, reduces lesion formation, and promotes granulation tissue formation [111]. Examples of some mechanical properties of chitosan and its derivatives that are crucial for wound healing and tissue engineering are shown in Table 4.

**Table 3:** Recent updates on the application of different chitosan-based formulations for wound healing in various types of wounds.

Type of Formulation	Active Compound	Size	Type of Wounds	Zeta Potential	Special Comments	References
Quercetin alginate/chitosan gel	Quercetin	-	Excision wound	-	In comparison to free quercetin, the developed gel was topically efficacious and showed synergistic wound healing potential in Wistar albino rats.	[112]
Chitosan nanofibres	Bromelain and silver nanoparticle	145 ± 73 nm	Second-degree burn	-	In this study, the authors revealed that the prepared bromelain-and-silver-nanoparticle-based CS nanofibres are a promising solution for wound healing.	[113]
CSNPs	*Dunaliella salina*	425.19 ± 4.21 to 496.89 ± 7.62 nm	Full-thickness excision lesions	22.51 ± 0.50 to 29.21 ± 0.33 mV	This is the first study to show that D. salina-loaded nanoparticles effectively cure wounds.	[5]
Chitosan nanofibres	*Cissus quadrangularis* (CQ)	77.9–97.4 nm	Cell line study	-	Bilayer sponges containing natural CQ extract exhibited encouraging outcomes as a prospective biomaterial for wound healing purposes.	[114]
CSNPs	Epidermal growth factor (EGF)	63.5–127 nm	Excision wounds	+35 to +40 mV	The developed chitosan nanoparticles are a viable medium for safe delivery of EGF for wound healing applications.	[115]
Chitosan hydrogels	Melanin nanoparticles	216 ± 30 nm	-	+30 ± 14 mV	This work proposes a unique three-dimensional therapy based on natural macromolecules that accelerates wound healing through dual routes while minimising the wound stress response, which is important for the development of therapeutic phototherapy techniques.	[6]
CSNPs	Gallic acid	252.90 ± 3.09 nm	Excision wound	+33.50 ± 0.30 mV	The hexosamine and collagen content were highest in the CSNP-treated group.	[116]
Hydrogel	Lupeol-loaded chitosan–Ag+ nanoparticle	291.9 ± 23.1 to 508.1 ± 26.9 nm	Infected full-thickness wounds	-	The developed hydrogel exhibits significant promise as a versatile therapeutic platform with the capacity to expedite wound healing and proficiently combat bacterial infections in clinical environments.	[117]
CSNPs	Curcumin	359 ± 65 nm	Full-thickness wound	−10.70 ± 0.10 mV	The curcumin-loaded-CSNP-treated groups show the highest collagen content.	[118]
Patches	Doxycycline	50–100 nm	Excision wound	– 24.4 mV	The authors reported that the chitosan-based skin patch incorporating doxycycline holds promise as a viable dressing for the management and enhancement of skin wound healing.	[119]
AgNPs	Ag	190–200 nm	Full-thickness wound	-	CS-AgNPs generated hydroxyproline content of 27.53 0.47 mg/g, 1.6 times greater than the control group and nearly identical to the level seen in the original tissue.	[120]
CS-AgNPs	Ag	200 nm	Excisional wound	-	The result from this study reveals that the prepared CS-AgNPs enhance the antibacterial and wound healing activity.	[121]
CSNPs	Melatonin	160–165 nm	Diabetic full-thickness wound	+25 Mv	No significant difference in collagen content was observed.	[122]
CSNPs	Curcumin	257–260 nm	Full-thickness wound	+30 ± 14 mV	The curcumin-loaded CSNPs hasten the wound healing due to synergistic action shown by chitosan and curcumin.	[123]
CSNPs	Insulin	294–300 nm	Full-thickness wound	+17.89 ± 0.74 mV	The prepared scaffold helps in wound healing; there was a 45% reduction in wound size.	[124]
CSNP-loaded nanofibres	Curcumin	32.17 ± 0.39 nm	Excisional wound	-	The prepared electrospun nanofibres show superior antibacterial and antioxidant properties. The nanofibres loaded with curcumin nanoparticles also help in wound healing.	[125]

### 4.4. The Recent Updates on the Wound Healing Activity of Chitosan

A previously published work describes the development of a new multifunctional wound dressing based on a carboxymethyl chitosan (CMC)/sodium alginate (Alg) hydrogel embedded with a simvastatin (SIM)-loaded nanostructured lipid carrier (NLC). This dressing’s design aimed to provide a multi-pronged therapeutic strategy by guarding against infections, controlling excess exudates, and speeding wound healing (Table 4). The scientists used a rigorous approach to composite manufacturing, optimising the CMC-to-sodium Alg ratio at 1:2. This optimised composite demonstrated admirable wound management characteristics, indicating its ability to take in exudates effectively while preserving an optimal moisture environment for the wound. The researchers synthesised a nanocomposite hydrogel using a freeze-drying method. The resultant composite has favourable properties such as a porous structure and a high swelling capacity, which are essential for good wound treatment. The authors have created a multifunctional wound dressing that combines the advantages of synthetic and bioactive polymers, nanotechnology, and a well-known medicinal drug, simvastatin. The encouraging in vitro findings emphasise the composite’s potential as a next-generation wound dressing for the treatment of diverse chronic wounds. More in vivo research is required to confirm these results and assess the dressing’s efficiency in a complex biological system [71,72,73,74,75,116,143].

Nie et al. (2023) [144] developed a groundbreaking self-healing and self-injecting chitosan hydrogel (QCS-ODex), addressing a critical gap in biopolymeric wound dressings. Leveraging the synergistic interaction between glycidyl trimethyl ammonium chloride-graft-chitosan (QCS) and aldehyde-dextran (ODex) in physiological conditions, the QCS-ODex hydrogel demonstrated exceptional characteristics: rapid gelation within 70 s, a porous morphology with 300–350 µm pore sizes, enhanced swelling capacity (2.465 times that of chitosan), superior tissue adhesion, and notable transmission, free radical scavenging, self-healing, injectability, and innate antibacterial properties against *E. coli* and *S. aureus.* Additionally, incorporating Baicalein allowed for modulated release based on QCS-ODex ratios, augmenting its antioxidative and antimicrobial efficacy. Formed through a Schiff base reaction under physiological conditions, these hydrogels exhibited promising potential for transparent, self-healing, and injectable applications in wound care, particularly suited for irregular and dynamic wounds. Their multifunctionality and capacity for real-time wound monitoring open new avenues for enhancing wound healing strategies [77,78,117]. The summary of research findings on chitosan and its derivatives are shown in Table 5.

### 4.5. Chitosan in Tissue Engineering

**Figure 5:**
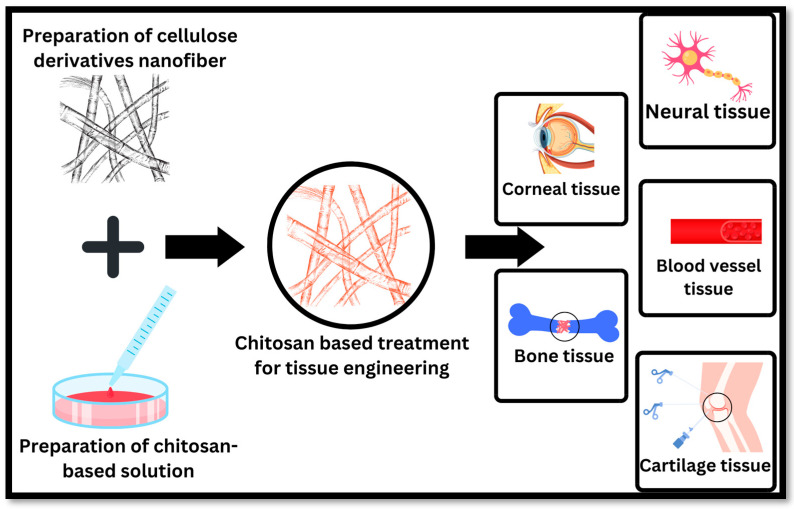
Applications of chitosan-based pharmaceutical preparations in tissue engineering include bone tissue, cartilage tissue, blood vessel tissue, neural tissue, and corneal tissue.

Using chitosan in tissue engineering has shown to be a viable and adaptable option. As a result of its biocompatibility and biodegradability, it is a promising scaffold material for use in regenerative medicine and tissue engineering. However, additional study is required to completely comprehend the potential of chitosan in tissue engineering and maximise its usage in therapeutic applications [156,157]. Chitosan is a scaffold used in tissue engineering to promote cell development and tissue regeneration. Films, fibres, and hydrogels are just a few examples of the many chitosan scaffold preparations that may be made to support cell growth and differentiation in three dimensions. The scaffold provides a temporary framework for supporting newly generated tissue until it can maintain itself [158,159,160]. Chitosan’s biocompatibility is a significant characteristic of scaffold material. Chitosan is well suited for use in tissue engineering due to its lack of toxicity and inability to provoke an immunological response. Cell adhesion, proliferation, and differentiation are all critical steps in tissue regeneration, and chitosan has been proven to stimulate all three [161]. Chitosan’s biodegradability is a notable characteristic as a scaffold material used in tissue engineering (Table 6). The risk of long-term side effects is lower with chitosan because enzymes gradually degrade it in the body. The products of this breakdown are eliminated normally. For this reason, chitosan is a promising scaffold material for procedures in which the scaffold will be gradually reabsorbed by newly created tissue [162]. A previously published study addressed the obstacles of regenerating oral and craniofacial bone defects, which can vary from minor periodontal and peri-implant problems to more severe and critical defects. Current therapies have limitations, highlighting the need for innovative techniques such as tissue engineering. A crucial element of successful bone tissue engineering is the design of a scaffold with specific properties, such as biocompatibility, biodegradability, mechanical strength, and osteoconductivity using chitosan [163]. Mesoporous silica nanoparticles (MSNs) were synthesised, and freeze-dried MSNs were incorporated into porous composite scaffolds made of alginate and chitosan. Various proportions of MSNs (10%, 20%, and 30%) were utilised. The incorporation of MSNs into scaffolds resulted in a significant increase in mechanical strength without reducing porosity significantly. All of the samples displayed favourable swelling characteristics, which are advantageous for cell attachment and growth. The presence of MSNs in scaffolds led to a concentration-dependent reduction in hydrolytic degradation. Importantly, the MSN-containing scaffolds exhibited no toxic effects on cell viability. Compared to the control group, the Alg/Chit/MSN30 scaffolds not only exhibited noncytotoxic properties but also significantly increased cell viability. In addition, the nanocomposite scaffolds containing MSN demonstrated greater biomineralisation properties than the Alg/Chit composite, indicating their potential for bone tissue engineering applications. The results suggest that Alg/Chit/MSN30 scaffolds hold promise for bone tissue regeneration and that mesoporous silica nanoparticles have significant potential in tissue engineering in addition to their extensive biomedical applications [164]. The research centred on the use of 3D printing to fabricate scaffolds for tissue regeneration. Collagen and chitosan composites are regarded as ideal scaffolding substances for tissue engineering, but their poor printing capability prevents their use in 3D printing. The objective of the study was to surmount this limitation by developing hybrid collagen/chitosan bioinks with enhanced printability via a hydrogen bond interface. By modulating the temperature, bioinks were printed into scaffolds with desirable rheological properties and gelation temperature. The results indicated that the addition of chitosan decreased the swelling ratio of the printed scaffolds, whereas the proportion of collagen increased the degradation rate. The increasing values of Young’s modulus and tensile strength indicate that chitosan has a strengthening impact. Cell viability tests revealed robust cell proliferation on the scaffolds’ surfaces, as well as cell migration within and at the base of the scaffolds. It was discovered that chitosan improves the printability of collagen, facilitating the fabrication of hybrid collagen/chitosan scaffolds with regulated properties that are suitable for various tissue engineering applications. The study demonstrated that hybrid scaffolds could combine the benefits of collagen and chitosan and have been utilised in a variety of tissue engineering applications (Figure 5). In addition, it addressed the difficulties associated with collagen solution as a bioink, such as the lengthy gelation time and low mechanical strength of the formed gels. Despite lacking temperature sensitivity and shear-thinning behaviour, chitosan enhanced the ability to print collagen, possibly as a result of increased hydrogen bonds between the two substances. Concerning swelling properties and degradation rate, the hybrid scaffolds exhibited comparable swelling ratios but differing degradation rates, indicating that chitosan played an essential part in structure retention due to its stronger molecular interactions. The scaffolds retained their three-dimensional structure, fostering cell proliferation and tissue regeneration. In contrast to pure collagen scaffolds, composite scaffolds did not diminish, most likely due to the lower-temperature printing and crosslinking techniques employed. It was discovered that the proportions of collagen and chitosan influence the degradation rate and mechanical properties of hybrid scaffolds, providing the possibility of control for various tissue engineering applications. Studies on cell migration demonstrated that viable cells could migrate to the bottom of the scaffolds. Cell migration was influenced by the surface features of the scaffolds, with more compact and smoother surfaces promoting cell migration. The structure of the scaffolds, particularly those with a greater proportion of chitosan, had an effect on the number of viable cells, potentially as a result of increased mechanical strength inhibiting cell migration. Additionally, gentler hydrogel structures were found to be more suitable for cell habitation. The 3D-printed collagen/chitosan scaffolds, which resembled the structure of the extracellular matrix (ECM), provided interconnected apertures and adequate nutrition for cell proliferation. The scaffolds’ controllable pore structure facilitated cell growth, migration, and nutrient transport, making them appropriate for applications in tissue engineering [165].

### 4.6. The Recent Updates on Chitosan in Tissue Engineering

A study introduced an innovative synthetic bone graft solution, addressing the limitations of autografts such as donor site discomfort, infection risks, and graft scarcity. This approach leverages regenerative medicine and tissue engineering principles, focusing on novel biomaterials that enhance bone regeneration and prevent bacterial infections, particularly in dental applications. The proposed method combines chitosan (CS) and hydroxyapatite (HA) fibres infused with doxycycline (DX), utilising an injection pump for fabrication. In vitro results showed controlled DX release influenced by HA content, with 1% CS displaying optimal stability, ease of manipulation, and appropriate cuttability and pH. The study highlights the potential of the CS-HA-DX complex as an effective graft material for dental bone tissue regeneration, offering a multifunctional controlled release system. However, further research is needed to refine parameters like HA particle size for improved injectability and to explore milder crosslinking conditions, potentially through polymer combinations. The study suggests the dual drug delivery capability of this complex as a promising avenue for future investigation, contributing significantly to regenerative medicine and tissue engineering by presenting a viable bone regeneration strategy [177].

Another study investigated an innovative approach for subchondral bone replacement, crucial for regenerating the bone–cartilage interface in joints damaged by various factors. Deep joint issues often compromise mobility and functionality due to affected subchondral bone. This research explores biomaterial-based solutions to enhance patient quality of life. It presents a novel strategy using drug- or bioactive-loaded porous tissue scaffolds composed of nano-hydroxyapatite (nHAp), chitosan (CS), and either hydroxypropyl methylcellulose (HPMC) or Bombyx mori silk fibroin (SF). Fabricated via freeze-drying, these scaffolds were characterised through Fourier-transform infrared (FTIR) spectroscopy, scanning electron microscopy (SEM), X-ray diffraction (XRD), energy dispersive X-ray (EDX), and X-ray fluorescence (XRF), demonstrating mechanical properties akin to cancellous bone. They exhibited the controlled release of therapeutic agents like triamcinolone acetonide (TA) or transforming growth factor-1 (TGF-1) and supported mouse preosteoblast MC3T3-E1 cell attachment and proliferation, indicating biocompatibility. Furthermore, RT-qPCR analysis of scaffolds with TA highlighted their potential in modulating inflammation and bone-specific biomarkers. This advancement in tissue engineering, particularly for subchondral tissue regeneration, underscores the potential of CS, nHAp, and HPMC or SF-based scaffolds as bone substitutes [178].

A new study discussed how chitosan-based materials can be used to for skin tissue engineering. This article makes a significant difference in the field of tissue engineering, especially in the area of skin wound repair. The authors make and test a new three-dimensional composite scaffold made of sulphated silk fibroin, chitosan, and hydroxyapatite (SSF/CS/HAP), which was concluded to be a promising tissue engineering treatment. The authors claim that the stable mechanical properties, biodegradability, and antibiotic properties of this new structure are its most important features. The electron microscope shows that the scaffold has an area size of 15–20 um. This suggests that the environment is favourable for cell invasion and blood vessel growth, which is a key part of successful tissue engineering. The high growth index (779%) is another sign that the scaffold is effective at absorbing fluids, which could help with managing wound exudates. Co-culturing of L929 cells in vitro and testing for cytotoxicity with the CCK-8 assay showed that the scaffold is receptive to cells and has low cytotoxicity. These results show that it could help cells stick together and make more copies of themselves, which are two important steps in wound healing and tissue repair. In vivo tests show that the scaffold has practical value by showing that rats with neck cuts that were treated with the SSF/CS/HAP scaffold healed faster. Also, the scaffold seemed to assist hair cells to grow faster and more fully, which suggests that it plays a role not only in healing wounds but also in recovering the function and appearance of the skin. In summary, the article underscores the potential of a novel SSF/CS/HAP scaffold in advancing skin tissue engineering. Its high biocompatibility, good ability to keep in wetness, low acidity, and positive effects on wound healing and tissue growth all point to its possible therapeutic use [179].

### 4.7. Chitosan in Delivery Systems for Anticancer Drugs

Chitosan has shown great potential in drug delivery studies for cancer. It has been shown that chitosan may be utilised as a vehicle for anticancer medications, ensuring that only cancer cells are affected by the treatment and that healthy cells are spared any potential harm. As a result, therapeutic effectiveness is increased while toxicity is decreased. Many medications, including those used in chemotherapy, small interfering RNA (siRNA), and plasmid DNA have been successfully delivered using chitosan-based drug delivery systems [180,181]. Cancer imaging is another area where chitosan has shown potential. For non-invasive cancer cell imaging, chitosan may be coupled with imaging agents like fluorescent dyes or radiolabelled chemicals. Radiolabelling can be used in diagnostic and follow-up therapy evaluations [182,183]. Cancer cell growth and proliferation are two processes that chitosan has been tested for inhibiting directly in research laboratories. In cancer cells, chitosan has been demonstrated to cause apoptosis, or programmed cell death, and to suppress angiogenesis, forming new blood vessels that fuel tumour development [184,185]. Although chitosan has shown some positive results in cancer studies, further research is required to grasp its potential and limits thoroughly. More study is required to evaluate the clinical efficacy of chitosan in cancer therapy because there is a disconnect between the outcomes of preclinical studies and the success of clinical trials. More research is required to completely comprehend the scope of chitosan’s promise and its limits in cancer treatment. In Table 7, studies related to chitosan used in cancer research are discussed. Published research has investigated the use of chitosan in the intravesical bacillus Calmette–Guerin (BCG) and interleukin-12 (IL-12) as treatments for superficial bladder cancer. Even though BCG has worked in some cases, a large number of individuals still experience treatment failure or the tumour comes back. The study suggested mixing IL-12 with chitosan, a biodegradable protein, to improve the release of IL-12 and make the bladder cancer treatment work better. A study on mice with orthotopic bladder tumours showed that chitosan/IL-12 has a higher chance of curing the cancer than either IL-12 alone or BCG. Also, the anticancer effects of chitosan/IL-12 were long-lasting and completely protected against a tumour coming back. Analysis of the cytokines in the urine showed that chitosan/IL-12 caused the body to make more TH1 cytokines than either IL-12 alone or BCG. Immunohistochemistry showed that T cells and macrophages had moved into the tumours after being treated with chitosan/IL-12. The bladder submucosae of mice that had been treated had small numbers of immune cells that slowly went back to normal over a few months. Researchers think that intravesical chitosan/IL-12 is a safe and useful treatment that should be studied more in human studies for treating superficial bladder cancer. The study results back up the idea that the unique qualities of chitosan can make IL-12 treatment for bladder cancer work better. The study showed that there are differences between injecting IL-12 and putting it under the skin, with the latter having higher cytokine levels. Analysis of cytokines in the urine and antibody staining indicate that chitosan/IL-12 works better than BCG and IL-12 on their own. Based on the results, the study showed that urine cytokines could be used to predict how well intravesical chitosan/IL-12 would work in clinical trials. The study additionally revealed that treatment with chitosan/IL-12 is well tolerated and does not cause any long-term changes that are harmful. However, the study claimed that local dosing of IL-12 mixed with chitosan is a potential method with a wider treatment window and less systemic harm. In the end, the study showed that intravesical chitosan/IL-12 might be a good way to treat superficial bladder cancer with immunotherapy. The results of the combination treatment are better than those of BCG and IL-12 alone in terms of life, the production of TH1 cytokines, and protection against the return of the tumour. The results indicated that intravesical chitosan/IL-12 should be studied more in clinical studies to treat bladder cancer that is close to the surface [186].

### 4.8. Recent Updates on Chitosan in Cancer Research

An intriguing study paper presented a hydrogel chitosan-based nanocomposite system for 5-fluorouracil (5-FU) distribution that shows promise for the treatment of breast cancer. It is a captivating examination of the usage of chitosan in cancer research. The authors have developed a nanocarrier called Chitosan/Agarose/-alumina/5-FU that consists of crosslinked chitosan and agarose, as well as alumina nanoparticles and 5-FU, and they highlight the advantages of this composite in promoting effective drug delivery, superior cell elimination, and minimised side effects. Robustness and biocompatibility are promised by the synthesis of the polymeric hydrogel using the crosslinking of naturally occurring polymers, chitosan, and agarose. The addition of alumina nanoparticles strengthens the mechanical properties of hydrogel while improving the effectiveness of drug encapsulation. In order to evaluate the properties and confirm the composition of the nanocarrier, the study makes use of a wide range of methods, including dynamic light scattering (DLS), a field-emission scanning electron microscope (FESEM), Fourier-transform infrared (FTIR), and X-ray diffraction (XRD). Notably, the nanocarrier’s pH sensitivity, which emerges under acidic circumstances as enhanced 5-FU release, raises the possibility that it is a good candidate for targeted drug delivery, thus reducing the systemic adverse effects of chemotherapy. The MTT test and flow cytometry results employing MCF-7 breast cancer cell lines show that the 5-FU-loaded nano-emulsion is much more effective at eliminating malignant cells than free 5-FU in vitro trials. The fact that the death of the malignant cells was mostly apoptotic rather than necrotic suggests that the 5-FU is the primary cause of the cytotoxic effects, indicating that the nanocarrier components have less direct cytotoxicity and are thus more appealing as drug delivery systems. In conclusion, the study offers a thorough examination of the possible applications of chitosan-based nanocomposites in the treatment of cancer. The authors highlight the possibility of such nanocomposites in cancer treatment by showing how the nanocarrier may increase the therapeutic efficacy of 5-FU while reducing its negative effects [197].

In a newly released study, chitosan-modified liposomes are thoroughly analysed as a potential delivery method for the natural antitumour compound curcumin (CUR). The work develops a CUR-loaded platelet membrane bioinspired chitosan-modified liposome (PCLP-CUR) for targeted cancer treatment, building on earlier research on platelet membrane-camouflaged nanoparticles. The formulation’s properties and behaviour have been thoroughly investigated by the authors, who also provide solid proof of its increased curcumin release and greater tumour targeting ability. It is shown that the biomimetic PCLP-CUR lipidic nanocarriers have great biocompatibility, better bioavailability, longer circulation time, and improved tumour specificity. The addition of chitosan, which not only enhanced drug release behaviour but also permitted quick cargo release under moderately acidic tumour settings, is particularly noteworthy. PCLP-CUR’s pH-responsiveness, which enables quicker and more complete drug release in acidic conditions, presents a viable strategy to lessen the negative effects of chemotherapy on healthy cells. Another important discovery was PCLP’s avoidance of macrophage absorption, which was linked to the CD47 protein on the platelet membrane. Furthermore, both in vitro and in vivo tests supported PCLP-CUR’s improved tumour targeting capacity. This is because P-selectin on the platelet membrane interacts with the CD44 receptor on HepG2 cells. Both in vitro and in vivo tests on mice with HepG2 tumours show that PCLP-CUR has higher anticancer activity, with PCLP-CUR therapy dramatically reducing tumour size. The alteration in chitosan may potentially hasten the release of drugs at tumour sites. Based on weight change, organ H&E staining, blood biochemistry analysis, and cytocompatibility studies, the safety profile of PCLP-CUR seems promising; this is probably because it uses natural polysaccharides and biocompatible cell membranes. This research offers a thorough academic assessment of a liposomal delivery system for curcumin modified with chitosan. The PCLP-CUR nanocarrier’s novel platelet membrane camouflage and chitosan modification combination show significant potential for improving curcumin administration and effectiveness in cancer therapy [198].

An interesting article presents fascinating research on a novel chitosan-based nanocarrier for lung cancer therapy, a critical topic considering the global prevalence and mortality rate linked with this disease. The researchers propose a challenging yet intriguing nano-drug delivery system that can be used as an anticancer treatment agent as well as an antibacterial strategy. The authors recognise the potential of curcumin, a well-studied anticancer drug, but also recognise the limitations of its direct administration, most notably its low bioavailability. To get around this, they encapsulate curcumin within noisome nanoparticles, which are then covered with a chitosan polymeric shell. The use of chitosan is strategic because it increases the bioavailability of the nano-formulation in the lung, which is important given the mucociliary clearance inherent in this organ. The inclusion of Rose Bengal (RB), a photosensitiser, within the nano-formulation is a novel component of this work. This allows the dual-drug-loaded carrier to not only work as a targeted chemotherapeutic agent but also to have antibacterial capabilities, which is a novel strategy given the alterations in lung microbiota associated with lung cancer progression. Although the study provided encouraging results, a more in-depth analysis would increase its validity. Furthermore, while the nanocarrier displayed high toxicity against lung cancer cells as well as antibacterial activity against Escherichia coli, the work is severely hampered by the lack of in vivo testing. This leaves unsolved questions about the nano-formulation biodistribution, pharmacokinetics, and potential off-target consequences, all of which are important for assessing its real-world use. In essence, the research underscores the promise of utilising chitosan-based nanocarriers for addressing lung cancer. Nevertheless, it also emphasises the need for additional in vivo investigations and a deeper comprehension of the manufacturing process to establish the practicality of this distinctive nanocarrier system for potential clinical trials in the future [199].

## 5. Research Gap and Future Direction

The applications of chitosan and its derivatives have made significant strides in various fields, including biomedical engineering, pharmaceuticals, food science, and environmental remediation. However, several research gaps persist, paving the way for future directions and advancements in this area.

One significant research gap lies in the elucidation of the structure–property relationships of chitosan derivatives. While numerous derivatives have been synthesised with tailored properties, a comprehensive understanding of how structural modifications impact material characteristics and performance is still lacking. Future research should focus on systematic studies to correlate chemical structures, such as degree of deacetylation, molecular weight, and types of functional groups, with mechanical, biological, and physicochemical properties. This knowledge will enable more rational design and optimisation of chitosan derivatives for specific applications, enhancing their efficacy and versatility. Moreover, there is a need for further exploration of chitosan-based materials in advanced biomedical applications, such as controlled drug delivery systems, tissue engineering scaffolds, and regenerative medicine. While significant progress has been made in these areas, challenges remain in achieving precise control over drug release kinetics, enhancing tissue regeneration capabilities, and promoting long-term biocompatibility. Future research efforts should focus on developing innovative strategies, such as nanotechnology-based delivery platforms, biofunctionalised scaffolds, and combination therapies, to address these challenges and translate chitosan-based materials from bench to bedside.

Furthermore, the scalability and cost-effectiveness of chitosan production and processing methods represent another research gap that needs attention. While chitosan is derived from abundant natural sources, such as shrimp shells and fungal cell walls, the extraction and purification processes can be resource-intensive and environmentally burdensome. Future research should explore sustainable and eco-friendly approaches for chitosan production, including enzymatic and microbial fermentation methods, as well as the optimisation of downstream processing techniques to reduce energy consumption and waste generation. Additionally, efforts to develop scalable manufacturing processes for chitosan-based materials will be crucial for their widespread commercialisation and accessibility.

## 6. Conclusions

In conclusion, this review comprehensively explored the diverse applications of chitosan in contemporary pharmaceutical science. The investigation into the anti-inflammatory and antibacterial properties of chitosan has generated substantial interest due to its potential in medicine. Factors such as concentration, delivery, dosage form, immune cell type, and microenvironment all impact chitosan’s dual nature of exerting both pro-inflammatory and anti-inflammatory actions. Furthermore, recent advances in chitosan derivatives, such as salicylhydrazide chitosan Schiff’s base (SCsSB) and salicylhydrazide chitosan (SCs), have demonstrated enhanced antibacterial and anti-biofilm activities that outperform conventional agents, with potential in a variety of applications in medicine such as wound dressing and the administration of medication. These results further the investigation of chitosan derivatives as versatile biological agents, highlighting its potential for tackling global health issues such as antibiotic resistance. Chitosan’s wound healing activity is linked to its remarkable biocompatibility, biodegradability, and non-toxicity, all of which enable its therapeutic effect. Chitosan promotes cell proliferation, blood vessel formation, and the synthesis of extracellular matrix, which involves fibroblasts, keratinocytes, and endothelial cells. Furthermore, chitosan has antibacterial characteristics, increases growth factor release, and has immunomodulatory qualities that control inflammation and help tissue healing. Furthermore, chitosan’s hydrophilic nature allows it to maintain a moist environment, aiding cell movement and fostering granulation tissue production. Overall, chitosan shows promise as a wound healing agent in a variety of medicinal dose forms. This review emphasised how chitosan-based materials have the potential to be a successful strategy for tissue engineering applications. The results highlight chitosan’s importance in promoting bone and skin tissue regeneration by illuminating its biocompatibility, regulated drug release, and beneficial effects on wound healing and tissue development. The research on chitosan-based nanocomposites for cancer therapy concludes by presenting a hydrogel chitosan-based nanocomposite system for 5-fluorouracil (5-FU) distribution, which shows promise in the treatment of breast cancer. Increased biocompatibility, bioavailability, extended circulation time, and higher tumour selectivity are only some of the benefits of the modified chitosan PCLP-CUR nanocarrier, which also displays enhanced curcumin release and improved tumour targeting capabilities. To summarise, the findings of this research highlight the significant potential of chitosan in the field of pharmaceutical science, emphasising the requirement for additional exploration to fully harness its benefits. Further research is necessary to clarify the underlying mechanisms and optimise the design of nanocarriers based on chitosan. The ultimate goal is to enhance the specificity and effectiveness of these carriers in targeted drug delivery systems, particularly in the field of oncology, where minimising adverse effects and achieving high precision are critical. At the same time, there are significant obstacles that must be overcome to guarantee uniformity in the physicochemical properties of chitosan derivatives and to address the scalability of chitosan derivatisation methodologies. However, these challenges have the potential to greatly improve the commercial viability and translational potential of chitosan-centric advancements in the pharmaceutical and biomedical fields.

## Figures and Tables

**Table 1:** Some common examples of the modification of chitosan chemical structure to produce a different type of chitosan with different therapeutic activity.

Derivatives	Importance	Modification	Characteristics	Application	References
Quaternised chitosan	The mucoadhesive characteristics increase proportionally with the degree of quaternisation. This is similar to the enhanced cationic nature of chitosan, which influences its interaction with negatively charged mucin, resulting in mucoadhesion.	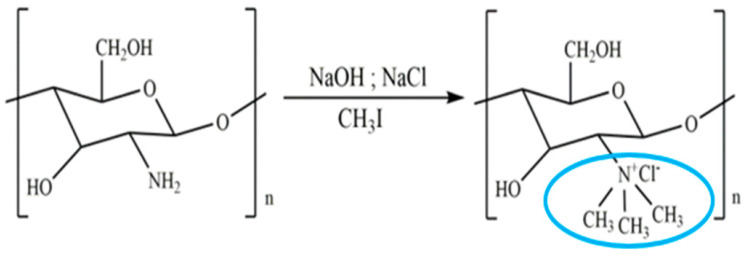	It helps various drugs to pass mucosal barrier and improves the water solubility and antioxidant activity.	Nasal vaccine adjuvant and antimicrobial agent.	[23,31,32]
Alkyl chitosan	It is proposed that the increased transfection efficiency of alkylated chitosan is due to increased cell entry facilitated by hydrophobic interactions, as well as easier unpacking of DNA from alkylated chitosan carriers due to the weakening of electrostatic attractions between DNA and alkylated chitosan.	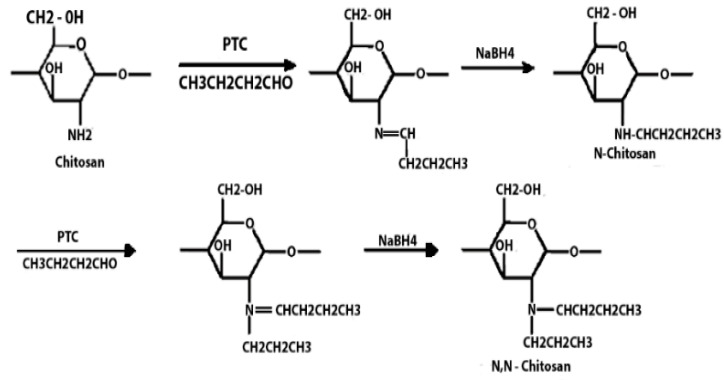	Improves transfection efficiency and forms clots with blood to improve coagulation efficiency.	Gene delivery and haemostatic dressing.	[23,33,34]
Highly cationic derivatives	Highly cationic derivatives of chitosan have been prepared, and their cationic nature is essential to many of their applications, including bio-adhesion, absorption enhancement, transfection efficiency, and biological activities like antitumour, antimicrobial, anti-inflammatory, and anti-hypercholesterolemic effects.	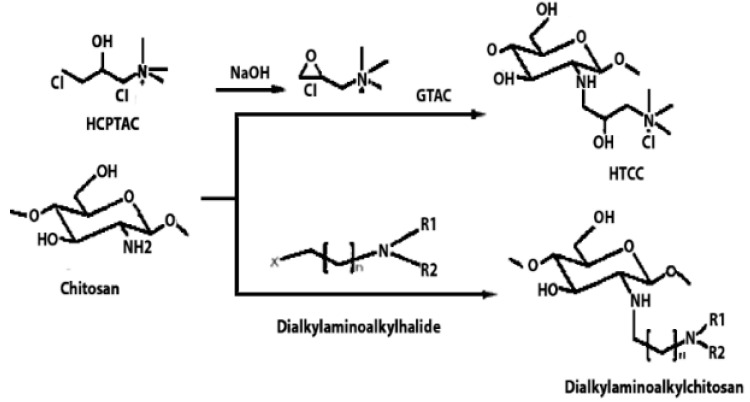	Enhanced water solubility, mucoadhesive properties, and improved interaction with negatively charged cell membranes.	Tissue regeneration and wound healing.	[35,36]
Hydroxyalkyl chitosan	Hydroxyethyl chitosan has great biocompatibility and biodegradability and is suitable for medicinal applications. It also has good bacteriostatic and hygroscopic moisturising properties and may be used as a natural textile softening and finishing agent.	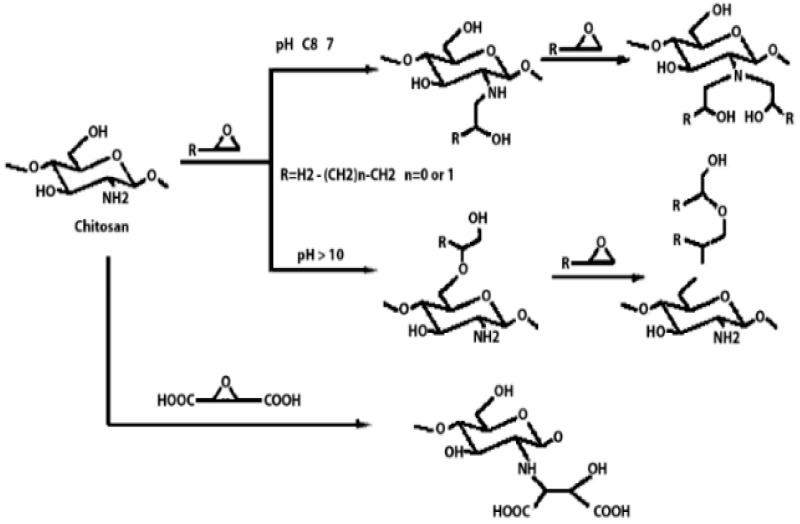	Improved mechanical strength and enhanced antibacterial activity and bioavailability.	Drug delivery carrier.	[37,38,39]
Carboxyalkyl chitosan	Carboxymethyl chitosan is a water-soluble chitosan derivative with antibacterial, anticancer, antitumour, antifungal, and antioxidant characteristics that is employed in both drug administration and enzyme delivery.	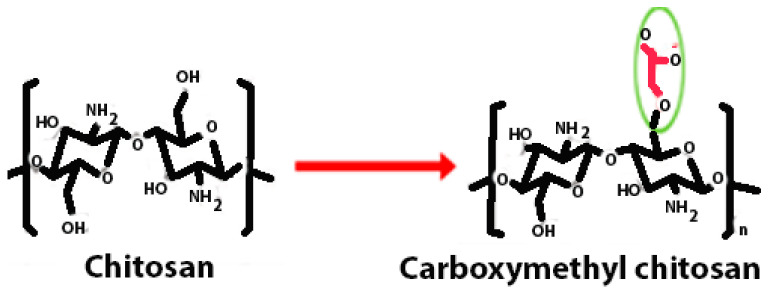	Improved water solubility and DNA binding capacity.	DNA delivery and site-specific protein delivery.	[40,41,42]
N-Acyl chitosan	N-acylated chitosan improves water solubility.	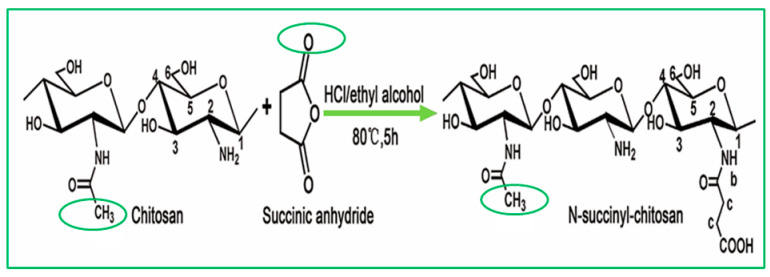	Improved encapsulation, stability, and adhesion.	Drug carrier.	[43,44]
O-Acyl chitosan	It improves its fat solubility and hydrophobicity.	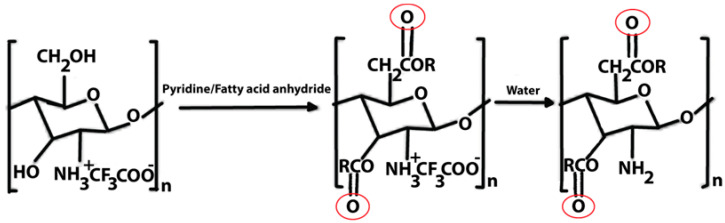	Improved adhesion and antibacterial activity.	Antimicrobial agent.	[45,46]
Thiolated chitosan	Thiolated chitosan offers various advantages over native chitosan, including increased solubility at low degrees of substitution and mucoadhesive and cellular penetration capabilities.	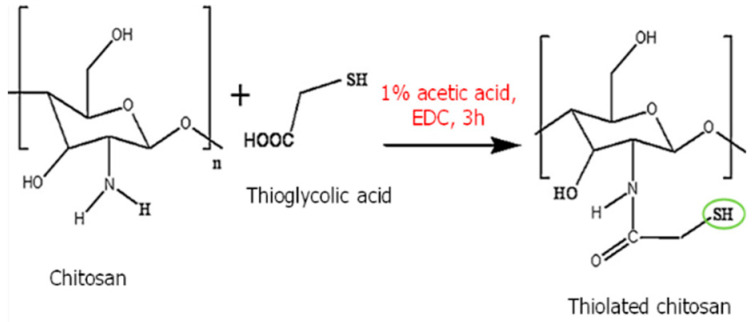	Increases the solubility and extends the release of drugs.	Drug carrier.	[47,48,49]
Azidated chitosan	Enhancement of mechanical stability.	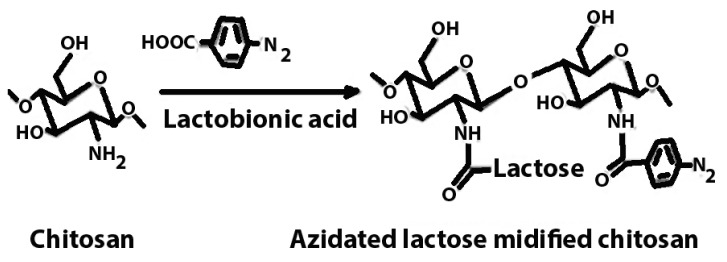	Enhanced mechanical strength, prolonged drug release kinetics, and targeted delivery.	Tissue engineering scaffolds in regenerative medicine.	[50,51]
Phosphorylated chitosan	Phosphorylated chitosan (PC), a water-soluble chitosan derivative, has various beneficial wound healing characteristics, including haemostatic capabilities, metal chelating capacity, antioxidant, anti-inflammatory, antibacterial, and angiogenic activity.	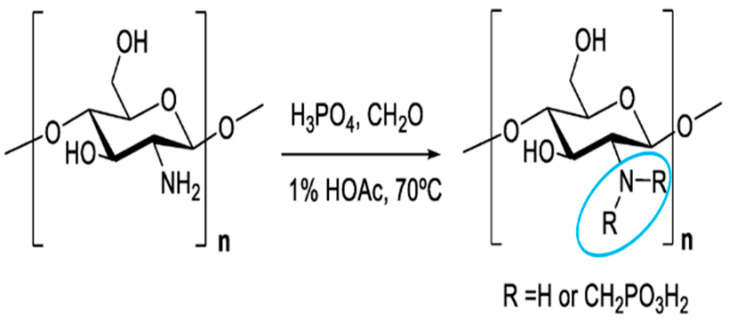	Promoting proliferation and osteogenic differentiation of MC3T3-E1s.	Bone tissue engineering.	[49,52]

**Table 4:** Mechanical properties of chitosan and its derivatives with the aim of wound healing and tissue engineering applications.

Chitosan Derivatives	Method of Preparation	Mechanical Properties	Special Comments	References
Trimethyl chitosan	Trimethylation	Mucoadhesion, tensile strength flexibility, and biodegradability.	Trimethyl chitosan tends to possess enhanced mucoadhesive properties compared to its unmodified counterpart, facilitating prolonged contact with tissue surfaces and providing a stable platform for drug delivery or tissue regeneration. Moreover, its increased water solubility and cationic charge may contribute to improved interaction with biological matrices, potentially enhancing cell adhesion, migration, and proliferation.	[126,127]
Carboxymethyl chitosan	Reductive or direct alkylation	Carboxymethyl chitosan combines the inherent biocompatibility and biodegradability of chitosan with the improved solubility and versatility conferred by carboxymethylation.	Carboxymethyl chitosan’s mechanical properties play a crucial role in facilitating the healing process. Its flexibility allows it to conform to the irregular contours of wounds, ensuring close contact with the wound bed and promoting a conducive environment for tissue regeneration. Chitosan’s adhesive properties enable it to adhere firmly to the wound site, preventing the displacement of dressings and promoting cell attachment and migration, which are essential steps in tissue repair.	[128,129]
Thiolated chitosan	Thiolation	Improved mechanical strength and elasticity compared to unmodified chitosan.	The modified form of chitosan, which incorporates thiol (sulfhydryl) groups, exhibits enhanced mucoadhesive properties, allowing for prolonged residence at the wound site or on mucosal surfaces.	[130,131]
Acylated chitosan	Acylation	Enhanced tensile strength, flexibility, and adhesion properties.	The mechanical stability of acylated chitosan materials ensures prolonged support to the healing tissue and promotes cell migration, proliferation, and tissue regeneration.	[34,132,133]
Alkylated chitosan	Alkylation	Enhance material’s flexibility, tensile strength, and elasticity.	The alkylated chitosan’s improved mechanical properties enable it to conform to wound contours, withstand mechanical stresses, and provide structural support during tissue regeneration processes.	[134,135]
Crosslinked chitosan	Condensation	Improved tensile strength, elasticity, and durability.	These enhanced mechanical properties enable crosslinked chitosan to withstand the dynamic mechanical stresses encountered in wound environments and tissue engineering constructs, providing stable support for tissue regeneration processes.	[136,137]
Glycol chitosan	Conjugation	Enhanced flexibility, biocompatibility, and biodegradability.	Glycol chitosan’s mechanical properties play a critical role in providing structural support to wound dressings and tissue scaffolds, facilitating cell adhesion, migration, and proliferation in the wound site or tissue defect.	[138,139]
Sulfoethyl chitosan	Alkylation	Enhanced adhesion, biocompatibility, biodegradability, and flexibility.	This derivative possesses enhanced mucoadhesive properties compared to unmodified chitosan, allowing for prolonged residence at mucosal surfaces, such as those found in wounds or damaged tissues. Its mechanical strength, flexibility, and adhesion properties enable it to conform to irregular wound contours, providing a protective barrier and promoting cell attachment, proliferation, and tissue regeneration.	[140,141,142]

**Table 5:** Summary of research findings on chitosan and its derivatives: This table provides an overview of the key materials, methodologies, preclinical findings, clinical relevance, and other significant aspects of studies focused on developing chitosan and its derivatives for enhanced antibacterial efficacy in wound healing applications. Each entry is identified by an article ID, facilitating easy reference to detailed study outcomes and potential clinical applications in skin regeneration and tissue engineering.

Ref.	Key Materials	Methodology	Preclinical Findings	Clinical Relevance	Bacterial Strains Tested	Antibacterial Mechanism	Safety and Biocompatibility	Future Research Directions
[145]	Chitosan-alginate and gentamicin	Electrospinning and in vitro and in vivo evaluations	Improved antibacterial activity, enhanced skin regeneration in Balb/C mice, and promotion of collagen deposition and formation of new blood vessels and hair follicles in treated wounds.	Potential use in preventing infections in wounds, specifically in skin regeneration and scaffolding for tissue engineering applications.	*Staphylococcus aureus* and *Escherichia coli*	Gentamicin inhibits protein formation, disrupts nucleic acid reproduction, and causes bacterial cell membrane rupture.	Demonstrated minimal cytotoxicity in L929 cell lines, supporting safe topical application.	Recommended studies on long-term effects, comparison with other antibacterial agents, and scaling up for clinical trials.
[146]	Chitosan, Ag nanoparticles, and LiOH/urea	Synthesis using LiOH/urea, characterisation (FTIR, XRD, SEM, TEM), mechanical testing, swelling studies, antibacterial activity studies, and wound healing studies in Sprague Dawley rats	Hydrogels showed ultrahigh mechanical properties with significant enhancement in compressive strength (15.95 ± 1.95 MPa). Demonstrated excellent antibacterial performance and accelerated wound healing with increased re-epithelialisation and collagen deposition compared to controls.	Could be used as an advanced wound dressing for skin regeneration, especially in scenarios requiring robust mechanical properties and high antibacterial activity.	*Staphylococcus aureus* and *Escherichia coli*	Silver nanoparticles disrupt bacterial membranes and prevent DNA replication, enhancing the antibacterial efficacy of the hydrogel.	Minimal cytotoxicity indicated in preclinical tests, suggesting good biocompatibility for topical application.	Further studies suggested for long-term effects and potential scaling up for clinical trials.
[147]	Chitosan, pectin, and TiO_2_ nanoparticles	Synthesis of nano-dressing, characterisation (FTIR, TGA, DSC, SEM, TEM), mechanical testing, antibacterial activity, and in vivo wound healing in albino rats	Nano-dressing enhanced wound healing evidenced by increased re-epithelialisation and collagen deposition. Showed good antibacterial properties and mechanical strength.	Potential for use as an advanced wound dressing material in medical settings due to its enhanced biocompatibility and effective wound healing capabilities.	*Staphylococcus aureus*, *Escherichia coli*, *Pseudomonas aeruginosa*, *Bacillus subtilis*, and *Aspergillus niger*	TiO_2_ nanoparticles disrupt microbial cell membranes, enhancing antibacterial activity.	Demonstrated minimal cytotoxicity and favourable blood compatibility, indicating safety for topical application.	Further studies recommended on long-term effects and evaluation in clinical settings.
[148]	PVA, chitosan, DMAEMA, and 1-bromobutane	Freeze-drying, crosslinking with glutaraldehyde vapor, FTIR, SEM, mechanical testing, antibacterial activity studies, and cytotoxicity and haemolysis tests	The prepared sponges showed improved water absorption (up to 2300%) and flexibility, significant antibacterial activity of nearly 100% against *E. coli* and *S. aureus*, excellent haemocompatibility, and cytocompatibility.	Ideal for advanced wound dressing applications due to its high porosity, enhanced mechanical properties, and effective antibacterial capabilities.	*Escherichia coli* and *Staphylococcus aureus*	Quaternary ammonium compounds in the sponge disrupt bacterial cell membranes, leading to high antibacterial efficacy.	Showed minimal haemolysis and cytotoxicity, indicating good compatibility for clinical use.	Further research could explore long-term clinical applications and effectiveness in diverse microbial environments.
[149]	Chitosan (CS) and S-nitrosoglutathione (GSNO)	Preparation of NO-releasing chitosan films (CS/NO), characterisation (FTIR, SEM, mechanical testing), antibacterial activity studies, and in vivo wound healing in rats	CS/NO films showed enhanced wound healing compared to CS films alone, with increased re-epithelialisation and improved histopathological outcomes. Strong antibacterial activity against *Pseudomonas aeruginosa* and *Staphylococcus aureus*.	Potential use as an advanced wound dressing for treating full-thickness wounds with enhanced antibacterial properties to prevent infections and promote wound healing.	*Pseudomonas aeruginosa* and *Staphylococcus aureus*	NO release from GSNO disrupts bacterial cell functions and structures, leading to antibacterial effects.	No adverse effects reported in in vivo studies, indicating good biocompatibility for topical application.	Suggest further studies on the long-term effects of CS/NO films and their clinical applications in human subjects.
[150]	Chitosan (CS), Dialdehyde Cellulose Nanocrystals (DCNCs), and silver nanoparticles (AgNPs)	Synthesis of AgNPs using periodate oxidation of CNC, forming of wound dressings via solution casting method, mechanical and antibacterial testing, and cytotoxicity assays	Enhanced mechanical properties and antibacterial activity of wound dressings; good cytocompatibility indicated by low cytotoxicity to NIH3T3 cells.	Potential for clinical application as advanced wound dressing materials due to enhanced durability, efficacy against infections, and safety.	Gram-positive and gram-negative bacteria and fungi including *Escherichia coli*, *Staphylococcus aureus*, *Klebsiella pneumoniae*, *Enterobacter cloacae*, *Streptococcus pneumoniae*, *Pseudomonas aeruginosa*, *Candida albicans*, *Candida glabrata*, and *Candida krusei*	AgNPs provide high antibacterial activity through interactions with microbial membranes, leading to cell damage.	Demonstrated minimal cytotoxicity and favourable biocompatibility for clinical use, supporting safe topical application.	Further research to explore long-term clinical effectiveness and potential for commercial scaling is suggested.
[151]	Chitosan (CS), Polyvinyl Alcohol (PVA), Polyvinylpyrrolidone (PVP), and Hexamethylene 1,6-di(aminocarboxysulfonate) (HMDACS)	Preparation of composite films using solvent casting method, characterisation (FTIR, SEM), mechanical testing, and antibacterial activity studies	Composite films showed improved mechanical properties, significant antibacterial activity, and optimal hydrophilicity for wound contact.	Potential for use as advanced wound dressings, particularly in settings requiring robust mechanical properties and effective antibacterial protection.	*Escherichia coli* and *Staphylococcus aureus*	Antibacterial activity attributed to the structured network of chitosan and its interaction with bacterial membranes, enhanced by PVA and PVP.	Demonstrated safety and minimal cytotoxicity, indicating suitability for clinical use in wound care.	Further research on long-term clinical performance and scalability of the production process.
[152]	Chitosan and nano-ZnO (nZnO)	Preparation of chitosan hydrogel/nZnO composite bandages, characterisation (FT-IR, XRD, SEM), and in vitro and in vivo evaluation	Composite bandages enhanced wound healing, showing faster re-epithelialisation and collagen deposition in vivo in Sprague Dawley rats. Demonstrated good swelling, cytocompatibility, and cell infiltration properties.	Promising for burn wounds, chronic wounds, and diabetic foot ulcers due to enhanced mechanical strength, flexibility, and significant antibacterial properties.	*Staphylococcus aureus* and *Escherichia coli*	Nano-ZnO provides antibacterial activity by damaging microbial cell walls and preventing microbial colonisation.	Showed minimal cytotoxicity and good biocompatibility, supporting its safety for clinical applications.	Explore long-term clinical effectiveness and potential customisation for specific wound care needs.
[153]	Chitosan and various additives for density and porosity control	Development and testing of chitosan-based wound coatings on male Wistar–Kyoto rats and rabbits, with evaluations including mechanical properties, biodegradation, and in vivo wound healing effectiveness	Coatings showed effective framework function, proper drainage, and supported rapid healing processes in purulent wounds. Specific coatings recommended based on wound type (purulent vs. granulating).	Promising for use in clinical settings for treating various types of wounds, particularly purulent wounds requiring robust and effective antibacterial barriers.	*Staphylococcus aureus*	Chitosan’s natural antibacterial properties enhanced by structural modifications to optimise contact with pathogens.	Demonstrated good biocompatibility with no adverse effects reported in animal tests, supporting potential for safe clinical use.	Further clinical trials to validate efficacy in human subjects and explore broader applications in wound care.
[154]	Chitosan and 3D printing technology	Preparation of 3D-printed chitosan scaffolds, in vitro cell growth studies, and in vivo wound healing tests on diabetic rats	Enhanced wound healing in diabetic rats; improved cellular adhesion and proliferation in vitro.	Potential application in diabetic wound management; showcases the effectiveness of 3D-printed scaffolds in regenerative medicine.	Not specified in the manuscript	N/A	Demonstrated biocompatibility with human skin cells, supporting safe application in medical treatments.	Suggest further studies to assess long-term effects and potential clinical applications in human diabetic wound care.
[155]	Chitosan, hyaluronic acid, and zinc oxide nanoparticles (ZnO NPs)	Fabrication of chitosan-based patches using solvent casting, characterisation (SEM, FTIR), antibacterial testing, haemostatic evaluation, and in vivo wound healing in rats	Patches showed excellent haemostatic properties and enhanced antibacterial activity and promoted faster wound healing in rat models.	Suitable for advanced wound care, particularly in managing bleeding and preventing infection in wounds.	*Staphylococcus aureus* and *Escherichia coli*	Zinc oxide nanoparticles disrupt bacterial membranes, enhancing the antibacterial properties of the patch.	Demonstrated biocompatibility and non-toxicity in animal studies, indicating safety for potential clinical use.	Further development for clinical trials and exploration of its efficacy across various wound types and patient demographics.

**Table 6:** Recent studies investigating the application of various carrier systems in tissue engineering, with specific focus on different tissue types, in vitro/in vivo tests conducted, and corresponding references.

S. No.	Carrier System	Tissue Type	In Vitro/In Vivo Tests	References
1.	Hydrogels	Neural tissue	PC 12 cell in vitro	[166]
2.	Nanocomposite matrix	Bone tissue	MG-63 cell line	[167]
3.	Injectable hydrogels	Bone tissue	MG-63 cell line	[168]
4.	Hydrogels	Cartilage tissue	-	[169]
5.	Scaffolds	Neural tissue	Rat pheochromocytoma (PC12)	[170]
6	Hydrogel composite scaffolds	Meniscus tissue	Rabbit mesenchymal stem cells	[171]
7.	Nanocomposite scaffolds	Skin tissue	Attenuated total reflectance–Fourier-transform infrared spectroscopy (ATR-FTIR)	[172]
8.	Three-dimensional (3D)-printed hydrogel scaffolds	Cartilage tissue	ATCD5 cells	[173]
9.	Injectable chitosan hydrogel	Cartilage tissue	The chondrogenic effect of bone mesenchymal stem cells (BMSCs) within the chitosan hydrogel was also assessed in vitro	[174]
10.	3D-printed scaffold	Bone tissue	MG-63 cell line	[175]
11.	Fibrous scaffolds	Bone tissue	The in vitro study showed that scaffolds fabricated at 90 °C promoted better MG63 cell attachment, proliferation, and differentiation	[176]

**Table 7:** This table presents a comprehensive overview of various carrier systems, including nanoparticles and nanofibres, along with their respective active compounds. The table highlights key findings such as size, entrapment efficiency, and notable observations from studies, emphasising the potential of these carrier systems for effective drug delivery in cancer therapy and other pharmaceutical applications.

S. No.	Carrier System	Active Compound	Size	Entrapment Efficiency	Key Findings	References
1.	Nanoparticles	Docetaxel	250.3 ± 1.7 nm	85%	The NP components were non-toxic and safe to human cells. The prepared nanoparticles may be used as effective carriers for chemotherapeutic agents targeting carcinogenetic tissues.	[187]
2.	Nanoparticles	Isolongifolene	200–250 nm	79.05 ± 4.60%	Isolongifolene-loaded chitosan nanoparticles were shown to be plasma-compatible and to have a consistent release pattern. As a result, chitosan-loaded nanoparticles might be used as an effective adjuvant in cancer therapy to address multi-drug resistance in solid tumours.	[188]
3.	Nanoparticles	Methotrexate	73.2 ± 4.9 nm	-	Methotrexate-loaded nanoparticles inhibited the viability of breast cancer cells when exposed to low doses. The tiny size of this efficient cytotoxic nanoparticle delivery method makes it a promising technique for use in breast cancer.	[189]
4.	Nanofibre	Cisplatin	200–350 nm	-	The prepared nanofibres are biocompatible and effective in cancer treatment.	[190]
5.	Nanoparticles	Docetaxel and curcumin	100–150 nm	7.82% and 6.48	The prepared nanoparticle loaded with curcumin and docetaxel can ameliorate the immunosuppressive microenvironment to promote the inhibition of tumour growth.	[191]
6.	Nanoparticles	Quercetin	291.1 ± 9.1	11.17 ± 1.12%	The prepared nanoparticles decrease tumour growth and provide a prospective strategy for the treatment of paclitaxel-resistant lung cancer.	[192]
7.	Nanoparticles	Doxorubicin	27.31 ± 0.78 nm	74.40 ± 1.60%	At pH 5.0, the drug was promptly and totally liberated from the nanoparticles. The anti-HER2 conjugated OCP copolymer nanoparticles had the lowest IC_50_ in vitro, indicating a boost in the therapeutic effectiveness of DOX to treat human breast cancer.	[193]
8.	Hydrogel nanocomposite	Curcumin	30 nm	62%	The proposed nanostructure is a promising vehicle with the ability to improve curcumin loading and enable prolonged curcumin release while causing considerable cytotoxicity in MCF-7 cells.	[194]
9.	Hybrid nanogel containing gold nanoparticles	Doxorubicin	119.3 nm	56%	The present research demonstrated that the designed enzyme-responsive nanogel could potentially target various solid tumours both actively and passively.	[195]
10.	Nanoparticles	Doxorubicin and Cisplatin	160 nm	21.4% for DOX and 81.71 for CP	In human breast cancer MCF-7 cells, DOX plus cisplatin had a synergistic cell-killing impact. The findings clearly show that the innovative nanoplatform holds great promise for synergistic combination treatment of breast cancer.	[196]

OCP—O-succinyl chitosan graft Pluronic^®^ F127; DOX—doxorubicin; CP—cisplatin.

## Data Availability

Not applicable.
